# Measuring appropriate need for unicompartmental knee arthroplasty: results of the MANUKA study

**DOI:** 10.1007/s00167-021-06632-6

**Published:** 2021-06-19

**Authors:** Antonio Klasan, Matthias Luger, Rainer Hochgatterer, Simon W. Young

**Affiliations:** 1 Department for Orthopaedics and Traumatology, Kepler University Hospital GmbH, Krankenhausstrasse 9, 4020 Linz, Austria; 2 Johannes Kepler University Linz, Altenberger Strasse 69, 4040 Linz, Austria; 3 North Shore Hospital, 124 Shakespeare Road, 0620 Takapuna, Auckland, New Zealand

**Keywords:** Unicompartmental knee arthroplasty, Total knee arthroplasty, Knee replacement, Usage, Appropriate use criteria

## Abstract

**Purpose:**

Indications for unicompartmental knee arthroplasty (UKA) are controversial. Studies based solely on radiographic criteria suggest up to 49% of patients with knee osteoarthritis (OA) are suitable for UKA. In contrast, the ‘Appropriate use criteria’ (AUC), developed by the AAOS, apply clinical and radiographic criteria to guide surgical treatment of knee OA. The aim of this study was to analyze patient suitability for TKA, UKA and osteotomy using both radiographic criteria and AUC in a cohort of 300 consecutive knee OA patients.

**Methods:**

Included were consecutive patients with clinical and radiographic signs of knee OA referred to a specialist clinic. Collected were demographic data, radiographic wear patterns and clinical findings that were analyzed using the AUC. A comparison of the radiographic wear patterns with the treatment suggested by the AUC as well as the Surgeon Treatment Decision was performed.

**Results:**

There were 397 knees in 300 patients available for analysis. Median age was 68 [IQR 15], BMI 30 [6] with 55% females. Excellent consistency for both the radiographic criteria and the AUC criteria was found. Based on radiological criteria, 41% of knees were suitable for UKA. However, when using the AUC criteria, UKA was the appropriate treatment in only 13.3% of knees. In 19.1% of knees, no surgical treatment was appropriate at the visit, based on the collected data.

**Conclusion:**

Application of isolated radiologic criteria in patients with knee OA results in a UKA candidacy is misleadingly high. AUC that are based on both radiological and clinical criteria suggest UKA is appropriate in less than 15% of patients.

**Level of evidence:**

III retrospective study.

## Introduction

Surgical options for management of knee osteoarthritis (OA) include joint preserving interventions, high tibial osteotomy (HTO) and distal femoral osteotomy (DFO), and arthroplasty procedures, unicompartimental knee arthroplasty (UKA), total knee arthroplasty (TKA), and patellofemoral arthroplasty (PFA) [18].

TKA is the treatment of choice for patients with bicompartimental or tricompartimental disease [18]. Younger, active patients with significant limb malalignment and preserved range of motion are candidates for an osteotomy [6]. These patients are also UKA candidates [6]. For the most common presentation, a patient with moderate to severe unicompartimental disease and minor changes in other compartments [21] controversy exists. The treatment options here are UKA and TKA [4]. UKA has an advantage of a lower cost [4] and a lower morbidity and mortality at the expense of a higher revision rate [25].

Higher UKA usage is associated with a lower revision rate, leading some authors to recommend that surgeons should increase their UKA usage [9, 15]. Reported UKA usage is only 10–15% in national joint registries [12]. Many authors argue that the percentage of knee OA patients suitable for UKA is much higher, at 47.9% [24] or even 49% [8]. Early reported UKA contraindications have been disproven and expanded [7], and current indications evolve mainly around radiographic patterns [8]. The only other published indication criteria are those of the American Academy of Orthopaedic Surgeons—the Appropriate Use Criteria (AUC) for surgical treatment of knee OA [3, 18] that incorporate both clinical and radiographic variables. The AUC were recently externally validated [2] and even though there are limitations to the AUC [20], criteria are useful in clinical practice as a helping tool and need to be evaluated.

The aim of this study was to analyze patient candidacy for TKA, UKA and osteotomy using published radiographic patterns and AUC in a cohort of 300 consecutive knee OA patients. It was hypothesized that AUC criteria will demonstrate a lower UKA candidacy than purely radiologic criteria within the same patient cohort.

## Materials and methods

### Patient cohort

This is a retrospective study, performed at a public tertiary referral center with a catchment population of approximately 700,000, where over 700 primary total knee arthroplasties are performed annually. TKA and osteotomies in the center are performed using computer-assisted surgery (OrthoMap, Stryker, Kalamazoo, MI, U.S.). Data were recorded on 300 consecutive patients referred for consideration for knee replacement surgery, between 01 September 2019 and 28 February 2020. The patients were assessed by one of 7 fellowship trained arthroplasty surgeons, each performing at least 50 cases of surgical knee OA management yearly. The public referral system is based on the patient's address with all referrals initially screened by one of the 7 orthopedic surgeons for the following criteria: length of the symptoms (> 6 months), daily use of analgesia for knee pain, at least moderate activity restriction, evidence of at least Grade I osteoarthritis according to the Ahlbäck classification [1] in any of the compartments and potential consideration for knee surgery. If all criteria are not met, the referral is declined and the patient is referred back to the GP for non-operative treatment. Patients with previous arthroplasty were excluded. Patients with inflammatory arthritis and with post-traumatic arthritis were not excluded, due to their presence in the daily practice of surgeons managing knee OA [10, 17].

### Data collection

The data collected were age, gender, body-mass-index (BMI), bilateral knee involvement and ethnicity [14]. Patients with symptomatic bilateral knee osteoarthritis were analyzed for each knee separately.

Each knee was assessed using antero-posterior weight bearing (AP), lateral in 45° of flexion and ‘Skyline’ patella views [24]. The referral, triage and clinical notes were retrieved for input into AAOS appropriate use criteria for surgical management of knee OA [3, 18], Table 2. The radiologic and AUC criteria retrieval and assessment was performed by two independent authors who were blinded to the subsequent Surgeon Treatment Decision made in the specialist clinic (RH, ML). The Surgeon Treatment Decision was added last to the database for analysis. The outcome was classified as TKA, UKA (medial or lateral), patellofemoral arthroplasty (PFA), bicompartmental arthroplasty or non-operative management/deferred with a planned follow-up. Surgeon Treatment Decision for UKA was guideline based [19], adhering strictly to minimal to no changes in any other compartments. Osteotomy was planned in patients < 60, with complete range of motion, unicompartmental disease and > 5 malalignment in the coronal plane [6]. Non-operative management was continued if the patient did not feel that the symptoms warrant surgery, after discussing this with the surgeon extensively. All other cases were planned for a TKA.

### Outcome measures

The radiographic wear patterns were classified according to the UKA candidacy criteria proposed by Willis-Owen et al. [24], Table 1. Wear patterns considered appropriate for UKA are knees with isolated anteromedial or lateral compartment wear, as well as anteromedial wear with medial PFJ wear [5, 24]. Patients who met these criteria were labeled “radiologic UKA candidates”. Patients with wear of both patellar facets, not described in the initial classification, were categorized in the categories with lateral wear: lateral PFJ (LP), anteromedial with lateral PFJ (ALP) and medial with lateral PFJ (MLP) (Table 1). A category combining lateral and lateral patellofemoral joint (LLP) compartment was added as it was commonly observed. Isolated medial patellofemoral and isolated lateral patellofemoral categories were merged into a single patellofemoral joint category (PFJ). If none of the wear patterns matched, the knees were radiologically classified as ‘other’.

**Table 1:** Modified patterns of knee arthritis, Willis-Owen [23] classification

Pattern	Definition
Anteromedial (AM)	Ahlbäck 1 or worse changes isolated to the medial compartment, anterior to the mid-sagittal plane on the lateral radiograph
Medial (M)	Ahlbäck 1 or worse changes isolated to the medial compartment, extending posterior to the mid-sagittal plane on the lateral radiograph
Lateral (L)	Ahlbäck 1 or worse changes isolated to the lateral compartment
Patellofemoral (PFJ)	Ahlbäck 1 or worse changes isolated to the patellofemoral joint compartment, any side
Tricompartmental (T)	Ahlbäck 1 or worse changes in all 3 compartments
Bicompartmental (B)	Ahlbäck 1 or worse changes in both tibio-femoral compartments
Anteromedial with medial PFJ (AMP)	A combination of both anteromedial and medial PFJ patterns
Anteromedial with lateral PFJ (ALP)	A combination of both anteromedial and lateral or both PFJ patterns
Medial with medial PFJ (MMP)	A combination of both medial and medial PFJ patterns
Medial with lateral PFJ (MLP)	A combination of both medial and lateral PFJ patterns
Lateral with lateral PFJ (LLP)	A combination of both lateral and lateral PFJ patterns
Other (O)	Not otherwise classifiable

The AUC criteria for TKA, UKA and osteotomy were then assessed [3], Table 2. After the input of the parameters, the AUC algorithm gives a rating for UKA, TKA and realignment osteotomy, rated as appropriate (score  7–9), may be appropriate ( 4–6) and rarely appropriate ( 1–3). Appropriateness according to the AUC is reported; however, for the purposes of a binominal analysis, cases that “may be appropriate” and “rarely appropriate” were grouped into not appropriate, and compared to “appropriate”.

**Table 2:** AAOS appropriate use criteria algorithm for surgical management of osteoarthritis of the knee

Indication profile	Answer options
Function-limiting pain	Function-limiting pain at moderate to long distances (walking moderate to long distances greater than one fourth mile)
	Function-limiting pain at short distances (limiting activity to two city blocks, the equivalent to walking the length of a shopping mall)
	Pain at rest or night
Range of motion extension/flexion	Full range of extension/flexion
	Lack of full extension (> 5 degree flexion contracture) and/or flexion < 110 degrees
	Lack of full extension (> 10 degree flexion contracture) and/or flexion < 90 degrees
Functional instability	No functional instability
	Functional instability
Pattern of arthritic involvement (medial tibiofemoral, lateral tibiofemoral or patellofemoral)	Predominantly one compartment
	More than one compartment
Imaging (joint space in most involved compartment)	Mild to moderate—joint space narrowing as visible on imaging
	Severe
Limb alignment	Normal alignment
	Varus/valgus
Mechanical symptoms (compatible with meniscal tear or loose body)	Mechanical symptoms
	No mechanical symptoms
Age	Young
	Middle-aged
	Elderly

The radiographic UKA candidacy were compared with the AUC criteria. The Surgeon Treatment Decision was compared with both the radiographic UKA candidacy and the AUC criteria and analyzed the factors that might affect the decision on the procedure.

The study was approved by the Waitemata District Health Board Research & Knowledge Centre (approval RM14701).

### Statistical analysis

Normality was tested using the Shapiro–Wilk test. Normally distributed data are presented with mean (± standard deviation), non-normally distributed data using median (interquartile range). The inter-observer agreement for radiological analysis and the clinical parameters between the two assessors was analyzed using the intraclass correlation coefficient (ICC), two-way mixed model. In cases where there was a discrepancy between the investigators, a third investigator was added and the case was resolved with a consensus between the three investigators. Continuous variables were compared using the Independent Samples *t*-test. Categorical variables were compared using the Chi-squared test, with Odds Ratio (OR) and 95% Confidence Intervals (CI) reported. Binominal logistic regression was performed in a single-step multivariate manner. The agreement of the Surgeon Treatment Decision with the AUC recommendations was expressed as a proportion. Power analysis was based on the least common procedure, osteotomy [2]. It was estimated that osteotomy will be appropriate in 5% or less cases, with an equivalence limit of 5%, with an alpha of 0.05 and a beta of 0.2, 326 cases were needed. SPSS v. 24.0 (IBM, Armonk, NY, US) was used for the data analysis. A *p* value of < 0.05 was considered statistically significant.

## Results

From the 300 included patients, there were 97 patients with bilateral symptomatic knee OA, giving a cohort of 397 knees for the analysis, Table 3. Seven patients (2.3%) had post-traumatic TKA, 3 patients (1%) had a previous osteotomy on the ipsilateral side and 16 patients (5.33%) had rheumatoid arthritis. The distribution of Surgeon Treatment Decision was: TKA in 68% of knees, UKA in 7.8%, surgical treatment was deferred in 23.1% of cases, PFA in 0.8% and there was one case of a bicompartmental knee arthroplasty, medial UKA and PFA, 0.25%.

**Table 3:** Demographic comparison between the “radiologic UKA candidates” group and “radiologic Non-UKA candidates” based on Willis-Owen^11^ criteria

Variable	Radiologic UKA candidates	Radiologic Non-UKA candidates	All patients	*p* value
Number (%)	*n* = 163 (41.1%)	*n* = 234 (58.9%)	*n* = 397	
Age,years, median (IQR)	67 (16)	69.5 (14)	68 (15)	**0.021**
Female gender, %	52.5%	64.1%	55%	**0.020**
BMI	30 (8)	31 (7)	30 (6)	**0.030**
Race	white 46.5%	53.5%	75.3%	**< 0.001**
	Asian 40.0%	60.0%	11.3%	
	Pacific islander 8.5%	91.5%	13.3%	
Surgeon Treatment Decision for UKA	17.2%	1.3%	7.8%	**< 0.001**

Variables with statistical significance are bolded

*UKA* unicompartmental knee arthroplasty

### Radiologic UKA candidacy

Based on the radiologic criteria of Willis-Owen et al. [24], 41.1% of knees were suitable for UKA (Table 3). The ICC for the radiologic criteria was 0.94. From the 97 patients with bilateral knee OA, 4 had a different wear pattern in each knee. The “radiologic UKA candidacy” group was younger, had a higher proportion of men, had a lower BMI and the highest proportion of white race, Table 3. The Surgeon Treatment Decision for UKA overlapped with 17.2% of radiologic UKA candidates. Multivariate regression analysis demonstrated that younger patients, lower BMI and white patients were more also likely to receive a UKA, Table 4.

**Table 4:** Binominal multivariate logistic regression with Surgeon Treatment Decision for UKA as the dependent variable

	Odds ratio	Wald	95% CI Lower	95% CI Upper	*p* value
Age	0.905	16.158	− 0.148	− 0.051	**< 0.001**
Gender	2.239	3.706	− 0.015	1.627	n.s
BMI	0.905	4.997	− 0.186	− 0.012	**0.025**
Race	0.096	5.448	− 4.315	− 0.376	**0.020**
Bilateral	1.149	0.110	− 0.681	0.958	n.s
Contralateral previous TKA	0.423	0.623	− 2.995	1.275	n.s
Posttraumatic/HTO	9.651e-8	1.990e-4	− 2260.746	2228.439	n.s

Variables with statistical significance are bolded

*UKA* unicompartmental knee arthroplasty, *TKA* total knee arthroplasty, *HTO* high tibial osteotomy

### Appropriate use criteria

The inter-observer agreement (ICC) for the AUC was 0.91. According to the AUC, TKA was appropriate in 74.3% of cases and “may be appropriate” in additional 24.4% of cases, Table 5. UKA was appropriate in 15.1% and “may be appropriate” in additional 38.3% of cases (Table 3). In a proportion of patients where UKA was deemed appropriate, the affected compartment was the PFJ. Since the AUC currently do not recommend an isolated PFA, UKA was appropriate in 13.3% of cases. Osteotomy was appropriate in 1 case (0.25%). Out of the 41.1% UKA candidates according to Willis-Owen [24] criteria, UKA was appropriate in 34.9% of those cases according to AUC criteria, Table 5.

**Table 5:**
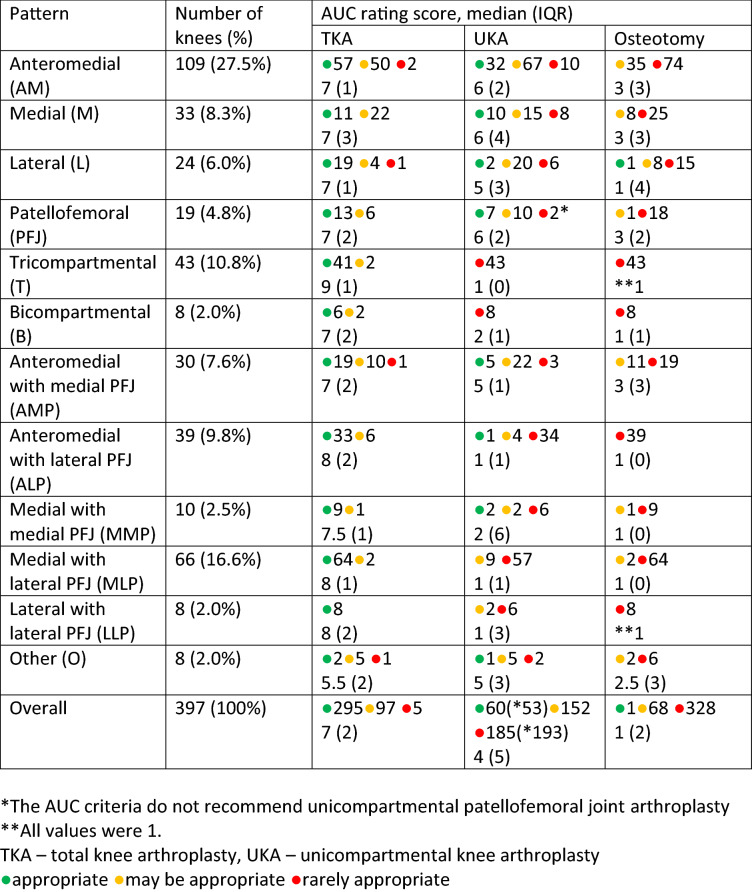
Distribution of radiological wear patterns, according to Willis-Owen [23] and the corresponding AUC criteria, for each pattern

Agreement between the Surgeon Treatment Decision with the AUC was the highest for TKA, Table 6. There were 24 cases where only UKA was appropriate, 2 of which were patients with PFJ arthritis. Half of these patients received no planned surgical treatment at the visit, Table 6. In patients where UKA and TKA was appropriate, the majority of patients received a TKA. In 76 knees (19.1%), the maximal score for any surgical treatment was 6, where no surgical treatment was deemed appropriate. Here, half of the patients received no surgical treatment, followed by TKA, Table 6. In no cases did the treating surgeon choose a ‘rarely appropriate’ treatment (scoring 1–3) according to AUC guidelines.

**Table 6:** The agreement between AUC and Surgeon Treatment Decision

Appropriate according to AUC	Number of knees (%)	Treatment decision (%)
TKA	259 (65.2%)	TKA 213 (82.%) UKA 9 (3.5%) No intervention 36 (13.9%) PFA 1 (0.4%)
UKA	24 (6.0%)	TKA 5 (20.8%) UKA 6 (25.0%) No intervention 12 (50.0%) Bicomp 1 (4.2%)
TKA and UKA	36 (9.0%)	TKA 22 (61.1%) UKA 7 (19.4%) No intervention 5 (13.9%) PFA 2 (5.6%)
Osteotomy	1 (0.25%)	TKA 1 (100%)
No surgical intervention	76 (19.1%)	TKA 29 (38.2%) UKA 9 (11.8%) No Intervention 38 (50.0%)

*AUC* appropriate use criteria, *TKA* total knee arthroplasty, *UKA* unicompartmental knee arthroplasty, *PFA* patellofemoral arthroplasty

## Discussion

The most important finding of the present study is the lower UKA candidacy of 397 patients with knee osteoarthritis, according to the AUC criteria, when compared to purely radiological criteria.

Using purely radiologic criteria, UKA candidacy has been reported as high as 47.9% of knees [24]. A more recently developed and validated radiological Decision Aid for UKA reported 49% of knees to be suitable for UKA [8]. This is consistent with the finding in the present study of 41% of radiological suitability for UKA. The presence of anteromedial OA (AMOA) is the primary indication for UKA, described as medial OA that does not extend to the dorsal aspect of the tibia on the lateral view as this indicates a functional ACL, of crucial importance for the mobile bearing UKA [8, 24]. The indication has also been expanded to include wear of the medial facet of the PFJ [5]. Although the candidacy in the present study was not as high as 47.9 or 49%, it was fairly close, 41.1.%, indicating similar wear patterns between the cohorts. Compared to the Willis-Owen et al. study [24], the present study observed a larger proportion of patients with an insufficient ACL, and secondly, a larger proportion of patients had lateral wear of the patellar facet, either isolated or in combination with medial wear. However, only 7.8% of patients received a UKA in the present study, which might be interpreted as bias towards TKA. It, however, reflects usage of UKA by the majority of knee surgeons [12]. Many authors declare such a percentage as underutilization of UKA, which in turn contributes to the higher reported UKA revision rates [9, 15]. Candidacy studies with high reported UKA candidacy do not take into account patients with rheumatoid arthritis, which is a contraindication for UKA, but is present in the daily practice [10, 17]. Even if patients with rheumatoid arthritis were to be excluded, the candidacy for UKA according to AUC criteria would increase in this study by 0.7%.

The high ICC for AUC in the present study is consistent with previous studies [2]. With the elimination of patients with PFJ OA as UKA candidates [3], the AUC analysis revealed only 13.1% of UKA candidates. Even in candidates for TKA and UKA, the TKA typically receives a higher rating (9) compared to UKA (7) [3], which is reflected in the present study. Decision aids and criteria are useful to a certain extent, however, experience, training and region all play a role in the final decision for the treatment of choice. Depending on the region, 10–15% of knee surgeons will reach the recommended threshold of 12 UKA/year or 20% UKA, and around 50% will perform less than 1% UKA [12].

One of the possible outcomes of the AUC is that no surgical treatment is “appropriate”, only “may be appropriate”. This outcome is reflected in the present cohort as well, with a 50% agreement with AUC. The deferral might lead to progression of OA in other compartments in a relatively short time, rendering an initially potential UKA candidate becoming a TKA candidate over time. Such outcomes are a part of the daily knee surgeon’s practice and demonstrate the complexity of the decision-making process, especially for UKA. Stating that such a high percentage of patients is suitable for UKA is misleading both for the surgeon and for the patient.

Although PFA is not recommended by the AUC in any setting, 3 surgeon treatment decisions for PFA and 1 for a bicompartimental UKA and PFA were observed. This expansion of indications is a consequence of the implementation of robotic assisted surgery, especially for UKA [11, 23]. Robotically assisted PFA has demonstrated promising early results [22] which may lead to further expansion of UKA or a combined arthroplasty. A recent study that assessed intraoperative wear patterns and ACL status of 300 patients found that two thirds of patients with end-stage knee OA could potentially be treated with partial or a combined partial knee arthroplasty [21]. Comparatively, in the present study, 52.9% of patients had medial or lateral wear and PFJ OA, could potentially be treated with a partial or combined partial arthroplasty, if ACL is intact. However, long-term data on the efficacy and cost-effectiveness of this treatment are limited.

### Limitations

This study has some limitations. The outcomes of the surgery were not evaluated, and it remains unclear which treatment option would have the best clinical results, and in many cases more than one treatment option may indeed have been appropriate. The study was, however, not designed to analyze the outcomes. If the UKA candidacy would to be expanded to include “may be appropriate” rating, the UKA candidacy would be 53.4%; however, the same expansion of criteria would deem TKA appropriate in 98.7% of cases. The demographic, socioeconomic, racial and other factors are region specific and complex [13]. The symptoms at presentation and the stage of the disease may, therefore, differ in different countries [16], but not in such a manner that would change the outcomes of the present study. The recently validated radiological decision aid for UKA [8] has not been utilized as the differences compared to the classification used differ only slightly and once again ignore the clinical findings.

### Conclusions

Application of isolated radiologic criteria for patients with knee OA results in a UKA candidacy that is misleadingly high. The AAOS Appropriate Use Criteria that are based on both radiological but mainly clinical criteria suggest that UKA is appropriate in less than 15% of patients.
